# Calcium Supplement by Tetracycline guided amorphous Calcium Carbonate potentiates Osteoblast promotion for Synergetic Osteoporosis Therapy

**DOI:** 10.7150/thno.45142

**Published:** 2020-07-09

**Authors:** Jianwei Wang, Shan Tao, Xiangyu Jin, Yanqing Song, Wentao Zhou, Haiya Lou, Rui Zhao, Cheng Wang, Fuqiang Hu, Hong Yuan

**Affiliations:** 1 College of Pharmaceutical Science, Zhejiang University, 866 Yuhangtang Road, Hangzhou, 310058, China.; 2 The Second Affiliated Hospital, School of Medicine, Zhejiang University, No. 88 Jiefang Road, Hangzhou, 310009, China.; 3 Sir Run Run Shaw Hospital, School of Medicine, Zhejiang University, No. 3 Qingchun East Road, Hangzhou, 310016, China.; 4 School of Pharmaceutical Engineering & Life Science, Changzhou University, Changzhou, 213164, China.

**Keywords:** calcium supplement, osteoporosis, amorphous calcium carbonate, osteoblast promotion

## Abstract

**Background:** The calcium supplement is a clinically approved approach for osteoporosis therapy but usually requires a large dosage without targetability and with poor outcome. This modality is not fully explored in current osteoporosis therapy due to the lack of proper calcium supplement carrier.

**Methods:** In this study, we constructed a tetracycline (Tc) modified and simvastatin (Sim) loaded phospholipid-amorphous calcium carbonate (ACC) hybrid nanoparticle (Tc/ACC/Sim).

**Results:** The resulted Tc/ACC/Sim was able to enhance its accumulation at the osteoporosis site. Most importantly, the combination of calcium supplement and Sim offered synergetic osteoblast promotion therapy of osteoporosis with advanced performance than non-targeted system or mono therapy.

**Conclusion:** This platform provides an alternative approach to stimulate bone formation by synergetic promotion of osteoblast differentiation using calcium supplement and Sim.

## Introduction

Osteoporosis is a common bone disease which can occur in any age as well as sex 1, 2. It is characterized as the disruption of bone homeostasis and loss of bone tissue which result in fracture and skeletal pain 3. The cause of osteoporosis remains unclear but clinical evidences have identified several beneficial treatments for osteoporosis, which include supplement of bisphosphonates, estrogen and related compounds, vitamin D and calcitonin 4. In particular, the calcium supplement in combination with vitamin D is supposed to be a conventional strategy for osteoporosis therapy 5. However, this approach usually requires relatively large dosage of calcium supplement and suffered from poor outcome which might due the fact that most of the adopted calcium ions are excreted without precise targeting to the lesions 6. Although nanomedicine employing advanced nanotechnology has shown beneficial effects on the reverse of osteoporosis 7-9, the calcium supplement modality for osteoporosis therapy is not fully explored in current osteoporosis therapies, which might due to the lack of proper calcium supplement carrier. Therefore, the development of osteoporosis targeted system might be challenging but highly desirable.

Our previous studies have revealed that amorphous calcium carbonate (ACC) as the unique form of calcium carbonate family usually exerts high water solubility upon exposure in aqueous environment while at the same reserves the acid responsive nature of common calcium carbonates, which results in faster calcium release of ACC in acidic environments as compared to other calcium containing materials 10, 11. In addition, it was generally recognized that the osteoclast activation is usually associated with the release of phosphate radicals, which gradually develops an acidic microenvironment within the osteoporosis lesions 12, 13. As a result, in our opinion, if being properly modified, ACC might be a preferable calcium supplement for osteoporosis therapy.

Tetracycline (Tc) is a broad-spectrum antibiotic which is employed to cure bacterial infections 14. However, in-depth study further reveals the preferable internalization of Tc into the bone, which possibly through a reaction that appears to occur directly between Tc and hydroxyapatite (HAP), the major constituent of bone tissue 15. It has previously been reported that bone disease may produce local inflammation that lead to blood exposure of HAP 16, which offers the possibility for targeting therapy of osteoporosis. As expected, several previous studies have suggested the promising bone targeting of Tc modified nanoparticles for osteoporosis therapy, which offers the possibility of employing ACC in osteoporosis targeted calcium supplement 17, 18.

The bone morphogenetic proteins (BMPs)-Smad is one of the important regulatory signaling pathway responsible for the formation of bone 19. It was well recognized that BMP, originated from the transforming growth factor (TGF)-β superfamily, plays an important role in the regulation of embryonic development 20. Employing the Smad signaling pathway to transduce osteogenic signals into bone marrow mesenchymal stem cells (MMSC), BMP not only promotes the differentiation of osteoprogenitor cells into osteoblasts, but also actives MMSC to differentiate into chondrocytes and osteoblasts 21, 22. Considering the fact that BMP down-regulation is usually observed in the progress of osteoporosis, accumulating evidences have revealed that simvastatin (Sim) can increase the expression of BMP-2 in osteoblasts and promotes the formation of new bone through the BMP-Smad signaling pathway 23, 24, which may be applied as a novel drug for the treatment of osteoporosis to break traditional methods of inhibiting osteoclast activity 4, 25.

**Scheme 1:**
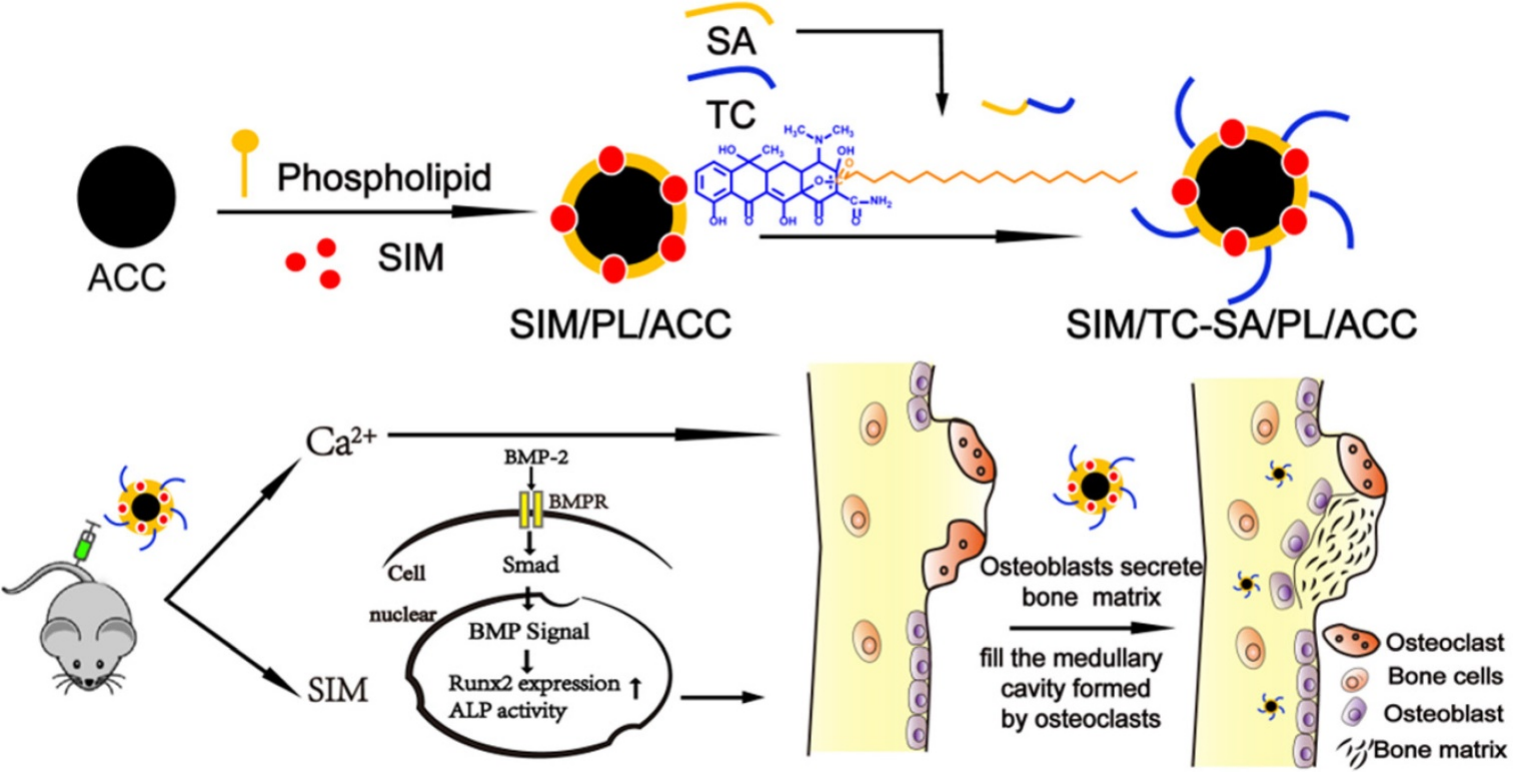
A biological mechanism by which calcium supplement using ACC augments Sim mediated BMP-2-induced osteoblast differentiation is shown as Runx2 expression and ALP activity through the BMP-Smad signaling pathway.

Herein our study, ACC nanoparticle was firstly synthesized as the core skeleton of the drug delivery system (DDS). Afterwards, the phospholipid (PL) was anchored on the surface of ACC to preserve the aqueous instability of ACC, during which the Tc conjugated stearic acid (Tc-SA) was inserted and Sim was loaded into the PL layer of the nanoparticles at the same time (Scheme 1). The finally constructed Tc/ACC/Sim was characterized regarding its size distribution, morphology, zeta potential as well as cytotoxicity on MC3T3-E1 (clonal newborn mouse calvaria) cells. The osteoporosis targeted capability of Tc/ACC/Sim was further evaluated and the optimized drug administration dosage was explored. Finally, the *in vitro* and *in vivo* osteoporosis reversing efficacy of Tc/ACC/Sim were investigated to confirm the promising osteoporosis therapy using synergetic calcium supplement and osteoblast promotion strategy. Our results concluded that calcium supplement in combination of Sim activated BMP-2 in osteoblasts (through the BMP-Smad signaling pathway) was suggested to promote the formation of new bone to finally reverse the osteoporosis process in treated subjects.

## Experimental Section

### Materials

Calcium chloride (CaCl_2_) and ammonium carbonate ((NH_4_)_2_CO_3_) were obtained from Shanghai Chemical Reagent Co., Ltd. (Shanghai, China). Hydroxyapatite (HAP, melting point: 1650 ℃; density: 3.16 g/cm^3^; water solubility: 0.4 ppm) was obtained from Sinopharm Chemical Regent Co., Ltd. (Shanghai, China). Phospholipid (PL, extracted from the egg yolk with the purity of ≥90%, CAS: 93685-90-6) was purchased from A.V.T. Pharmaceutical Co., Ltd (Shanghai, China). Sim was obtained from Zhejiang Hisun Pharmaceutical Co., Ltd. (Zhejiang, China). Otcadecylamine (ODA), 4',6-Diamidino-2-phenylindole (DAPI), fluorescein isothiocyanate (FITC), stearic acid (SA), N,N'-disuccinimidyl carbonate (DSC), 1-(3-dimethylaminopropyl)-3-ethylcarbodiimide (EDC), 1-Hydroxybenzotriazole (HOBT), L-ascorbic acid, alizarin red S, β-glycerophosphate and 3-(4,5-dimethylthiazol-2-yl)-2,5-diphenyl-tetrazolium bromide (MTT) were obtained from Sigma (St.Louis, MO, USA). Poloxamer 188 was obtained from Shenyang Jiqi Pharmaceutical Co., Ltd. (China). The bicinchoninic acid (BCA) protein assay kit and alkaline phosphatase (ALP) test kit were obtained from the Solarbio (Beijing, China). Indocyanine green (ICG) was purchased from Molecular Probes (Eugene, OR, USA). Alpha-minimum essential medium (αMEM) was from Thermo-Fisher Scientific (Waltham, MA, USA). Other reagents otherwise stated were of analytical and from Shanghai Chemical Reagent Co., Ltd.

### Synthesis and characterization of Tetracycline stearic acid graft (Tc-SA)

Firstly, Tc-SA was synthesized by a simple esterification reaction between hydroxy group of Tc and carboxyl group of SA in the presence of EDC and HOBT. Briefly, SA (213 mg), EDC (216 mg) and HOBT (150 mg) at the mol ratio of 1:1.5:1.5 (SA: EDC: HOBT) were co-dissolved in 30 mL anhydrous DMF. The solution was stirred at 40℃ with a mechanical stirring at 400 rpm for 30min to activate the carboxyl group of SA under the protection of nitrogen. Then, 10 mL anhydrous DMF containing 469 mg Tc (SA:Tc=1:1.3, mol:mol) was then added dropwise to the reaction solution. After 24 h of reaction under stirring at 40℃, Tc-SA was obtained, which was then dialyzed against distilled water for 48 h using a dialysis membrane bag (molecular cutoff = 7 kDa). After centrifugation at 4,000 rpm for 10 minutes and washed three times with the deionized water, final Tc-SA product was obtained, the structure of which was evaluated by ^1^H NMR.

### Preparation of fluorescein

We synthesized the fluorescent label ODA-FITC through a chemical grafting between the amino group of ODA and the isothiocyanate group of FITC according to our previous reported protocol 26. Briefly, 28 mg of FITC and 20 mg of ODA were dissolved in 6 mL of ethanol and stirred at 50 °C for 24 h in the dark. After which, 50 mL of distilled water was added to precipitate ODA-FITC. The product was washed three times with distilled water. In the end, the ODA-FITC was dried at room temperature and stored in the dark.

### Preparation of blank PL/ACC and Sim-loaded PL/ACC (PL/ACC/Sim)

ACC was firstly synthesized with a vapor-diffusion method 27. Briefly, 200 mg CaCl_2_ was dissolved in 300 μl distilled water, which was added to a glass bottle containing 100 mL absolute ethanol. The glass bottle was subsequently covered by parafilm with several pores. The bottle was put in a desiccator along with two glass bottles containing 3 g of solid (NH4)_2_CO_3_ at 35 °C. After vapor diffusion reaction for 1-2 days, the products were centrifuged (Allegra 64R, Beckman Coulter, USA, 15000 rpm, 10 min), rinsed several times and then re-dispersed in proper amount of absolute ethanol.

The ACC were then dispersed into ethanol containing PL (PL: ACC = 1, w/w) and 20% (w/w to PL) of Tc-SA (Tc/ACC) or not (PL/ACC). After stirring at 37 °C for 24 h, the mixture was ultrasonicated at 600 W for 20 cycles (20 KHz, 13 mm, work 2 s and stand 3 s) by a Lab ultrasonic cell pulverizer (JY92-II, Ningbo Scientz Biotechnology Co., Ltd, China) and then centrifuged at 3000 rpm for 10 min to remove unbounded free PL. The obtained supernatant was injected into water (1:9, ethanol : water ratio) under mechanical stirring. When preparing PL/ACC/Sim, Sim was added to the equivalent lipid component of blank PL/ACC (1:3 w/w, Sim : PL ratio). The other steps are the same as above.

### Characterization of Tc/ACC/Sim

First, the carrier concentration of ACC, Tc/ACC, PL/ACC/Sim and Tc/ACC/Sim were diluted to 100 μg/mL. Then, the hydrodynamic diameter and zeta potential were measured by DLS using the Zetasizer (3000HS, Malvern Instruments Ltd., UK). The morphological examination was further performed by transmission electron microscope (TEM, JEM1230, JEOL, Japan). Briefly, the samples were placed on a copper mesh, air dried naturally and then subjected to TEM observation.

### Drug loading content

The drug loading content of PL/ACC/Sim and Tc/ACC/Sim were analyzed by a high-performance liquid chromatography (HPLC) with an Agilent ZORBAX SB-C18 column (4.6 mm×150 mm, 5 µm). The constant flow rate was 1 mL/min and the column temperature was 35 °C. What is more, the detection ultraviolet wavelength was at 239 nm. The formulated PL/ACC/Sim and Tc/ACC/Sim dispersions were destroyed by adding a mixed solution (1:1 ratio of 0.6 M HCl : ethanol). Then, Sim was separated by centrifugation at 20000 rpm for 15 min. The concentration of free Sim in the supernatant was measured. The drug loading (DL%) of Sim were calculated using the following formula:

DL% = (W_0_ -W_a_)/(W_0_ -W_a_ +W)×100%

W_0_ is the total weight of drugs added to the system, W_a_ represents the weight of free drug in the supernatant, W represents total weight of the system.

### MC3T3-E1 cell culture

Clonal newborn mouse calvaria MC3T3-E1 cells were used as model cells to explore the behavior of nanocarrier in normal cells, which were cultured in αMEM medium containing 10% (v/v) FBS. When osteoblast differentiation and mineralization experiments were performed, 10 mmol/L β-glycerophosphate and 50 μg/L L-ascorbic acid were added to the basic medium. In addition, the cell culture medium was changed every other day 28.

### *In vitro* cellular uptake and *in vivo* bone targeting of Tc/ACC

MC3T3-E1 cells were seeded at 1×10^5^ cells/well in 12-well plates and incubated for 24 h until they were attached to the wells. Subsequently, cells were treated with fresh medium containing ODA-FITC labeled PL/ACC and Tc/ACC. To prepare the ODA-FITC labeled PL/ACC and Tc/ACC, ODA-FITC was added to the equivalent lipid component of blank PL/ACC and Tc/ACC (ODA-FITC: PL =1:10, w/w). Then ODA-FITC labeled PL/ACC and Tc/ACC were prepared as mentioned above. At predetermined intervals (2, 4, 6, 10 h) the cells were collected and observed under a confocal microscope (Olympus, Japan). In addition, semiquantitative analysis of cellular uptake was assessed with the flow cytometry (FC500MCL, Beckman Coulter, USA).

All animal experiments were approved by the Animal Care and Use Committee of Zhejiang University in accordance with the guidelines for the care and use of laboratory animals. The bone-targeting ability of PL/ACC and Tc/ACC was evaluated in ICR mice. The fluorescence dye ICG was used to detect the behavior of PL/ACC and Tc/ACC, which was loaded into the PL during drug loading (same as FITC labeling). Six ICR mice were randomly divided into two groups, three mice per group. ICG labeled PL/ACC and Tc/ACC were injected into mice through the tail vein. After 48 h, the mice were sacrificed, and their main organs (heart, liver, spleen, lungs, and kidneys) and the femur and tibia of the hind leg bones were obtained. The fluorescence intensity of the obtained organs and legs were subsequently detected with a Maestro EX *in vivo* imaging system (Maestro, USA) and calculated as a percentage using the following equation:


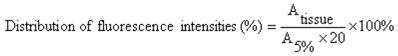


A_tissue_ is the fluorescence intensity of the major organs, and A_5%_ is the fluorescence intensity of ICG labeled PL/ACC or Tc/ACC at an injected dose of 5%.

### *In vitro* bone targeting of Tc/ACC

To investigate the bone mineral binding ability between bone-like substrates and bone-targeted delivery system *in vitro*, hydroxyapatite (HAP) assay was performed according to the previous report 29, 30. In brief, the initial fluorescence intensity of ODA-FITC labeled PL/ACC and Tc/ACC was measured (Ex [excitation wavelength] =496 nm, Em [emission wave-length] =523 nm, slit [width of slit] =5 nm, U [voltage of the photomultiplier] =700 V) using a fluorescence spectrometer (F-2500; Hitachi Ltd, Tokyo, Japan). Then, 20 mg HAP was added to the ODA-FITC labeled PL/ACC and Tc/ACC (2 mL aqueous solution, 1 mg/mL). The suspensions were stirred under room temperature with genteel mechanical stirring for 1 hour to allow the adsorption of the ODA-FITC labeled PL/ACC and Tc/ACC onto the HAP. The amount of the ODA-FITC labeled PL/ACC and Tc/ACC adsorbed was determined by measuring the difference between the fluorescence intensity of the initial stock solution and the supernatant after adsorption, according to the following equation:

HAP absorption affinity = (I_0_-Ia) / I_0_

where I_0_ represents the initial fluorescence intensities of the initial stock solution and Ia represents the fluorescence intensities of the supernatant after 1 hour adsorption.

### Optimization of administration dosage

We firstly evaluated the toxicity of the constructed nanocarriers (PL/ACC and Tc/ACC) to cells using MTT method. Afterwards, the Sim loaded DDSs were tested under different given drug concentration to explore the optimal concentration of Tc/ACC/Sim for osteoblast differentiation and mineralization. MC3T3-E1 cells were seeded at 1×10^4^ cells/well in 96-well plates and incubated for 24 h, after which cells were exposed to a series of concentrations of PL/ACC, Tc/ACC, Sim, PL/ACC/Sim and Tc/ACC/Sim for 48 h, after which 20 µL of 5 mg/mL MTT was added to each well for another 4 h followed by adding 200 µL of DMSO into each well to dissolve the forming formazan crystals. Subsequently, these plates were stirred at 37 °C for 30 min, and the absorbance of each well at 570 nm was measured by an automatic microplate reader.

### *In vitro* osteoporosis reversing effect

MC3T3-E1 cells were seeded at 1×10^5^ cells/well in 12-well plates and cultured in osteogenic differentiation and mineralization medium, which included Tc/ACC, Sim, PL/ACC/Sim and Tc/ACC/Sim (the concentration of Sim was 10^-7^ mol/L and the concentration of Ca^2+^ of Tc/ACC was the same as Tc/ACC/Sim) for 14 days. After 14 days of culture, the cells were fixed with 70% ethanol for 1 h, and subsequently stained using alizarin red S at the concentration of 1.5% at pH 4.0-4.2 for 10-15 min. Unreacted dye was removed and the formed mineralized nodules were observed with a light microscope. Finally, 10% cetylpyridinium chloride was added to each well to dissolve the alizarin red S in the cell matrix, and the concentration of alizarin red S was calculated by measuring the UV absorbance of the sample at 562 nm.

Besides, MC3T3-E1 cells were cultured in osteogenic differentiation and mineralization medium which includes Tc/ACC, Sim, PL/ACC/Sim and Tc/ACC/Sim as mentioned above for 7 days. After 7 days of culture, the MC3T3-E1 cells were lysed with PBS with 0.1% Triton X-100 under ultrasound. Afterwards, ALP activity was measured using an ALP test kit.

### Western blotting assay

MC3T3-E1 cells were seeded in plates and cultured in osteogenic differentiation and mineralization medium containing PL/ACC (the concentration of Ca^2+^ was 0.25 μg/mL). After 3 days of culture, MC3T3-E1 were lysed by the addition of cold RIPA lysis buffer and subjected to western blotting assay as reported in our previous study using corresponding antibodies (p-Smad and BMP-2) 31.

### *In vivo* osteoporosis reversing effect

Female Sprague Dawley rats, which had a mean body weight of 180-200 g were housed in a climate-controlled environment with a 12/12 h light/dark cycle with access to standard food and water.

Thirty female Sprague Dawley rats were randomly divided into six groups with five mice in each group: the SHAM group, the OVX group, the TC/ACC group and the Sim group, the PL/ACC/Sim group, the Tc/ACC/Sim group with a Sim dose of 1 mg/kg/2 days. All groups except the SHAM group were all bilaterally ovariectomized, whereas the SHAM group was subjected to a sham operation of abdominal invasion. The experimental groups were subjected to intravenous administration starting 1 month after bilateral ovariectomy, whereas the SHAM and OVX groups were administered saline. After 2 months of administration, all animals were sacrificed. The main organs (heart, liver, spleen, lungs and kidneys) and the femurs of rats were obtained. The excised organ samples (heart, liver, spleen, lungs and kidneys) of every animal were stained with H&E, after which they were observed with a light microscope (Nikon, ECLIPSE Ni).

Micro-CT was employed to analyze some changes in the trabecular bone of the femur. A SkyScan-1176 (Bruker micro CT, Belgium) system (18 μm voxel size, 90 kV, 278 μA, 230 ms exposure time, Al 0.5 mm filter, 180° rotation step) was used for the examinations. The 1.6 version of NR econ software (Bruker micro CT, Belgium) was used for 3D reconstruction and viewing of images. After 3D reconstruction, the 1.13 version of CT software (Bruker micro CT, Belgium) was used for bone analysis. The index included bone mineral density (BMD), bone volume fraction (BV/TV), thickness (Tb. Th), number (Tb. N) and separation (Tb. Sp) were all calculated to evaluate the bone formed in the defect area.

As mentioned earlier, Sim has the potential to increase the activity of BMP-2 and promote the formation of new bone *in vitro* and in animals through the BMP-Smad signaling pathway. During this process, ALP activity increases and tartrate-resistant acid phosphatase (TRAP) activity decreases. To evaluate the pharmacodynamic effect of Sim, all femurs were decalcified in EDTA, after which the femur were dehydrated and then embedded in paraffin. Then, sections (4 µm thickness) were stained with ALP and TRAP.

Besides, all femurs were decalcified in EDTA, dehydrated, and then embedded in paraffin to obtain sections of 4 µm thickness, which were then stained with hematoxylin and eosin (HE). In addition, the osteogenic effects of Sim and Ca^2+^ were assessed by immunohistochemistry (IHC) analysis of osteopontin (OPN) and osteocalcin (OCN). To further explore the mechanism of bone formation, IHC staining of BMP-2 was also performed.

## Results and Discussion

### Characterization of Tc/ACC/Sim

**Figure 1:**
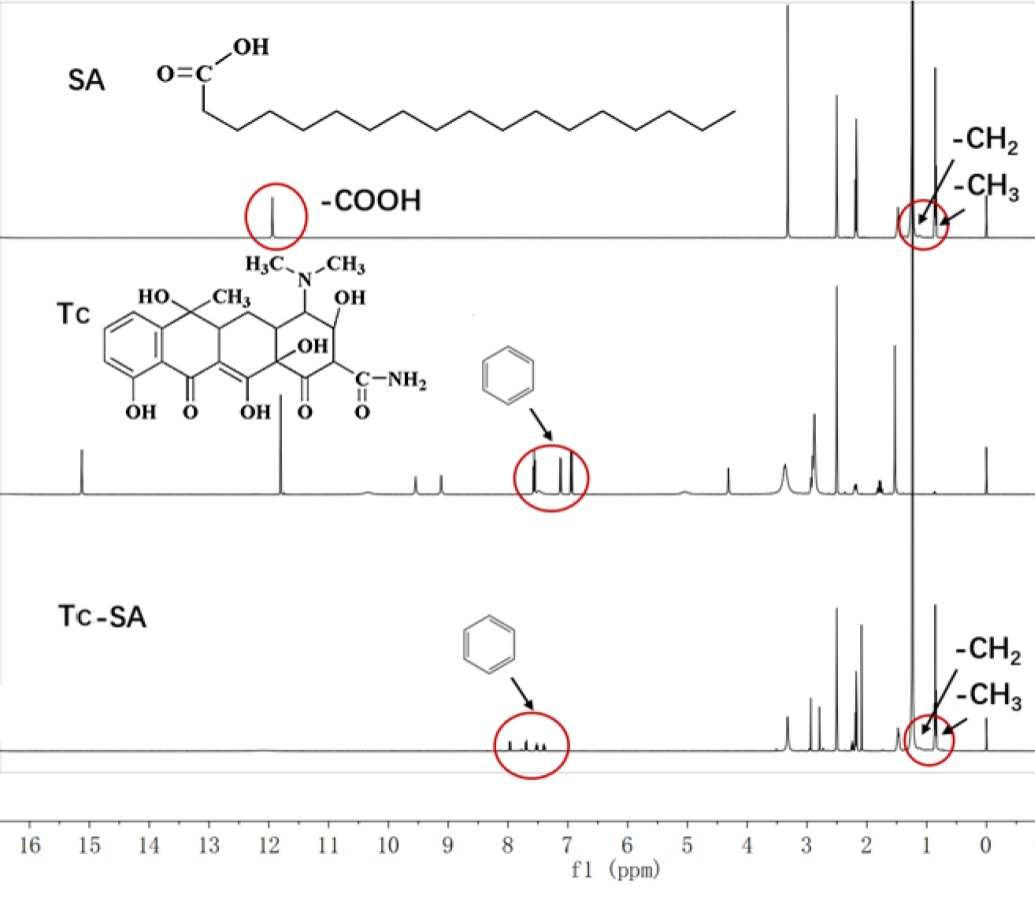
Characterization of Tc-SA using ^1^H NMR. Inserted images were corresponding chemical structure and peak assignments.

The Tc-SA as a key targeting moiety was firstly synthesized using chemical reaction. The carboxyl group from SA and the hydroxyl group from Tc were conjugated using EDC and HBOT as the catalyzers. The finally obtained Tc-SA was characterized using ^1^H NMR. As displayed in Figure 1, the peaks at 0.96 ppm and 1 ppm corresponded to the -CH_3_ and -CH_2_ of SA, respectively. In addition, the peak of -COOH from SA was also observed at 13 ppm. Accordingly, peaks at 7.0-8.0 ppm were assigned to the hydrogens of the benzene ring of Tc. As expected. The peaks of -CH_3_ and -CH_2_ from SA as well as peaks of the benzene ring from Tc were all clearly shown in the spectrum of Tc-SA while the peak of -COOH was disappeared. These results strongly suggested the successful preparation of Tc-SA through esterification reaction between the carboxyl group of SA and the hydroxyl group of Tc. The conjugation degree of Tc on SA was calculated to be 53.48% according to the results of ^1^H NMR.

**Figure 2:**
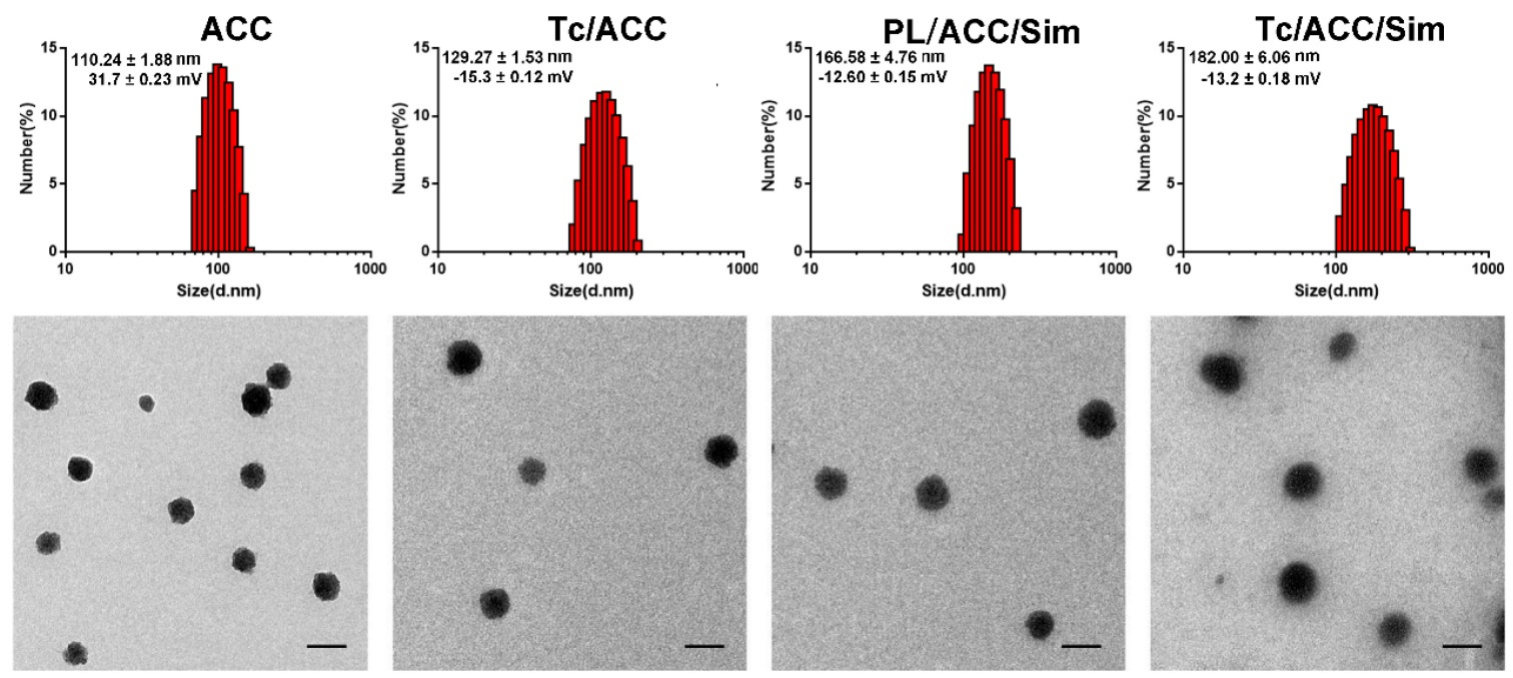
Characterization of Tc/ACC from ACC using (A) dynamic light scattering (DLS) and (B) transmission electron microscopy (TEM) images. Scale bar: 100 nm.

Afterwards, the preparation of Tc/ACC/Sim from ACC was investigated by monitoring the particle size distribution, morphology as well as zeta potential. As shown in Figure 2A, the ACC prepared using vapor-diffusion method was composed of numerous nano-sized particles with Z-average diameter around 110.24 ± 1.88 nm with a surface charge of 31.7 ± 0.23 mV. The surface modification of PL and Tc slightly increased the size of Tc/ACC to around 129.27 ± 1.53 nm (Z-average) with a significantly reversed surface charge to -15.3 ± 0.12 mV. The increase in size and the reverse in surface charge strongly indicated the successful anchoring of PL and Tc-SA on the surface of ACC, which was beneficial for the preserving of the water responsive nature of ACC and the targeting to osteoporosis tissues. After loading of Sim, the size of the obtained Tc/ACC/Sim further increased to 166.58 ± 4.76 nm (Z-average) with a constant surface charge of -13.2 ± 0.18 mV, which suggested that the Sim was loaded into the PL layer of Tc/ACC instead of being adsorbed on the surface of the nanoparticles. The Tc-SA unmodified PL/ACC/Sim was also constructed to investigate the effect of modification on the change in size and surface charge. The results (Figure 2A) revealed that the particle size of PL/ACC/Sim (182.00 ± 6.06 nm, Z-average) was similar to that of Tc/ACC/Sim with a comparable surface charge of -12.6 ± 0.15 mV, indicating that Tc-SA modification showed minor influence on the nanoparticle formation. The morphology observation using TEM was in Figure 2B. The results revealed that ACC was spheroid nanoparticle with independent boundary to one another. The following modification of PL and Tc-SA, as well as drug loading, showed insignificant influence on the morphology of the nanoparticles. As a result, the finally obtained Tc/ACC/Sim was well dispersed spheroid nanoparticle at the size range of around 100 nm, which was beneficial to maintain stable in the circulation for better osteoporosis therapy.

The linear relationship of the HPLC method adopted in our study for Sim determination was y=287.47x-53.604 (R^2^=1) with lowest detectable limit of 1.0 µg/mL. The DL of PL/ACC/Sim and Tc/ACC/Sim was 8.80 ± 0.30% and 7.90 ± 0.38%, respectively, which were sufficient for the osteoporosis therapy.

### *In vitro* cellular uptake and bone targeting of Tc/ACC

**Figure 3:**
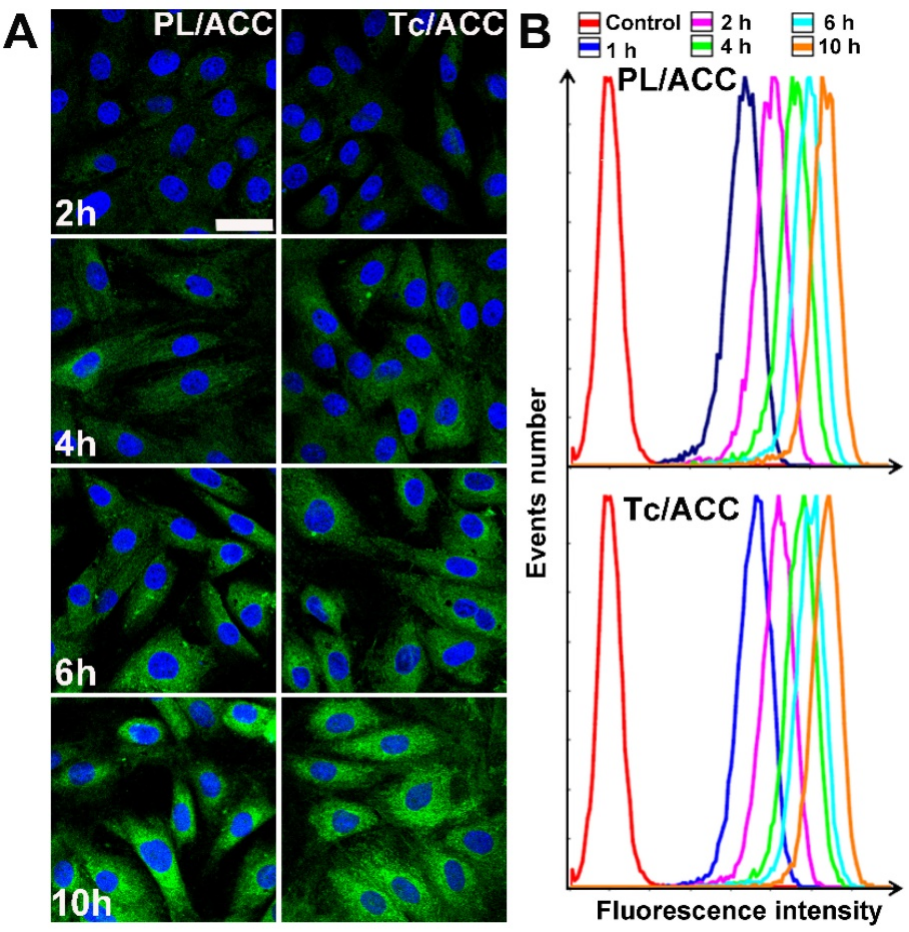
*In vitro* cellular uptake of Tc/ACC. MC3T3-E1 cells were incubated with ODA-FITC labeled PL/ACC and Tc/ACC for different time intervals (2, 4, 6, 10 h) and then subjected to observation using confocal microscope. (**A**) Time-dependent cellular uptake of ODA-FITC labeled PL/ACC and Tc/ACC. Scale bar: 30 µm. (**B**) Semiquantitative analysis of cellular uptake by flow cytometry.

ODA-FITC was synthesized and inserted into the PL layer of Tc/ACC as the probe to indicate the location of the DDS. MC3T3-E1 cells were treated by Tc/ACC or PL/ACC with or without Tc modification to explore the cellular uptake profile of the nanoparticles. As shown in Figure 3A, green fluorescence signal can be readily observed within MC3T3-E1 cells at merely 2 h post incubation and extended incubation resulted in further increased fluorescence signal in cells, which suggested the preferable internalization of both nanoparticles in MC3T3-E1 cells. Moreover, it was observed that although there were some variations in cellular uptake during the tests, compared to that of PL/ACC group, the Tc modification did not significantly change the uptake of Tc/ACC into MC3T3-E1 cells as time extended, which was also supported by the semiquantitative analysis using flow cytometry (Figure 3B). Further cellular uptake investigation using drug loaded nanoparticles revealed similar results (Figure S1). All results suggested that instead of specifically targeting the HAP in bone tissue, the Tc modification exerted minor influence on the endocytosis of nanoparticles into MC3T3-E1 cells which might be beneficial for the Sim delivery in the targeted cells after accumulation in the bone tissue. Moreover, the Tc/ACC with similar size and surface charge to that of PL/ACC might be suitable to serve as the carrier in the following studies.

**Figure 4:**
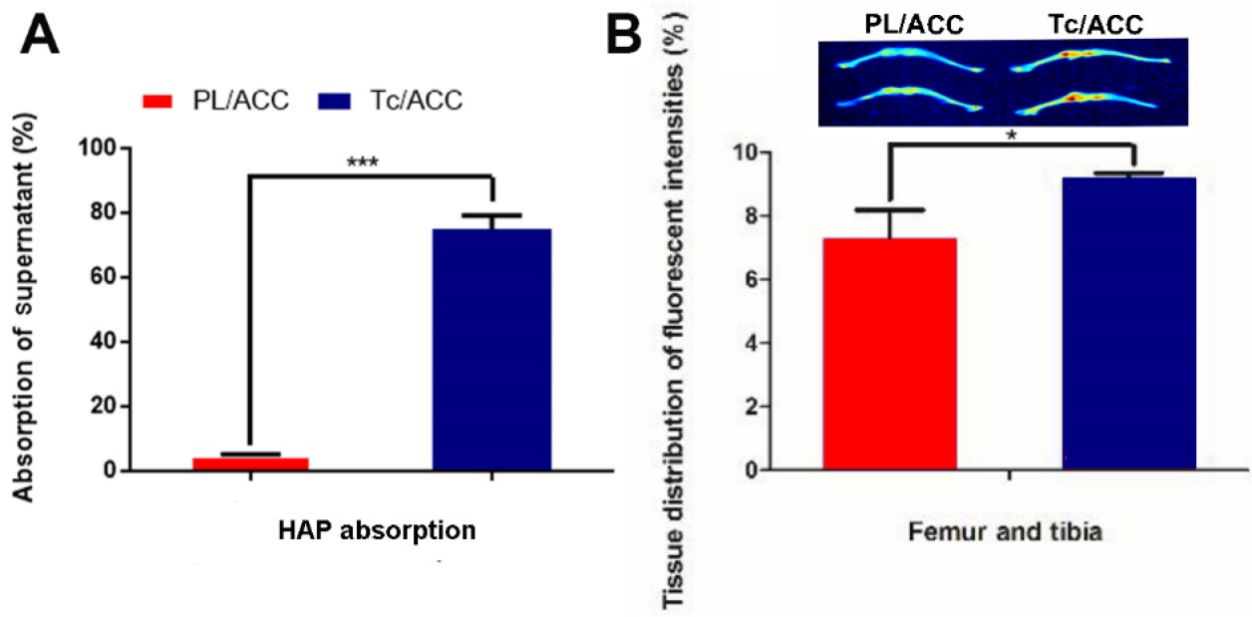
Bone targeting of Tc/ACC. (**A**) *In vitro* bone targeting of Tc/ACC using HAP adsorption assay. HAP was added to the ODA-FITC labeled PL/ACC and Tc/ACC and incubated for under genteel mechanical stirring for 1 hour. Afterwards, the decrease in the fluorescence of the supernatant was measured. (**B**) *In vivo* bone targeting of Tc/ACC using fluorescence imaging. ICR mice were randomly divided into two groups and intravenously injected with ICG labeled PL/ACC and Tc/ACC. At 48 h post administration, the mice were sacrificed and the quantitative accumulation of fluorescence signal in the femur, and tibia bones were calculated. Inserted image was fluorescence images of the distribution of ICG labeled PL/ACC and Tc/ACC in the femur and tibia bones. Results were expressed as mean ± S.D. (*n* = 3).

The *in vitro* bone targeting of Tc/ACC was firstly investigated using HAP absorption assay. The HAP was employed to simulate the exposed bone tissues in osteoporosis site. As shown in Figure 4A, after 1 h of incubation, significant difference in fluorescence intensity was found between the supernatant of PL/ACC and Tc/ACC groups. In detail, almost 75% of the Tc/ACC was absorbed by the HAP while the percentage drastically dropped to merely 4.7% in PL/ACC group, which strongly suggested the preferable binding of Tc to HAP for enhanced bone targeting.

Afterwards, the *in vivo* bone targeting of Tc/ACC in normal ICR mice was also investigated using ICG as the probe. It was noted that the fluorescence intensities in the bone of the Tc/ACC group were obviously higher than those in the PL/ACC group (inserted image in Figure 4B). These results suggested that the Tc modification was beneficial for the targeted accumulation of Tc/ACC to the bone tissue, which was favorable in osteoporosis therapy.

In order to further confirm this conclusion, the fluorescence intensities in bone tissues were quantified by corresponding software. As shown in Figure 4B, 25.72% more Tc/ACC was detected in the femur and tibia than the PL/ACC group. Both the *in vitro* and *in vivo* results confirmed that Tc/ACC could target bone tissue and accumulate at bone sites, which suggested the potential of Tc/ACC for bone-targeted delivery.

### Optimization of administration dosage

**Figure 5:**
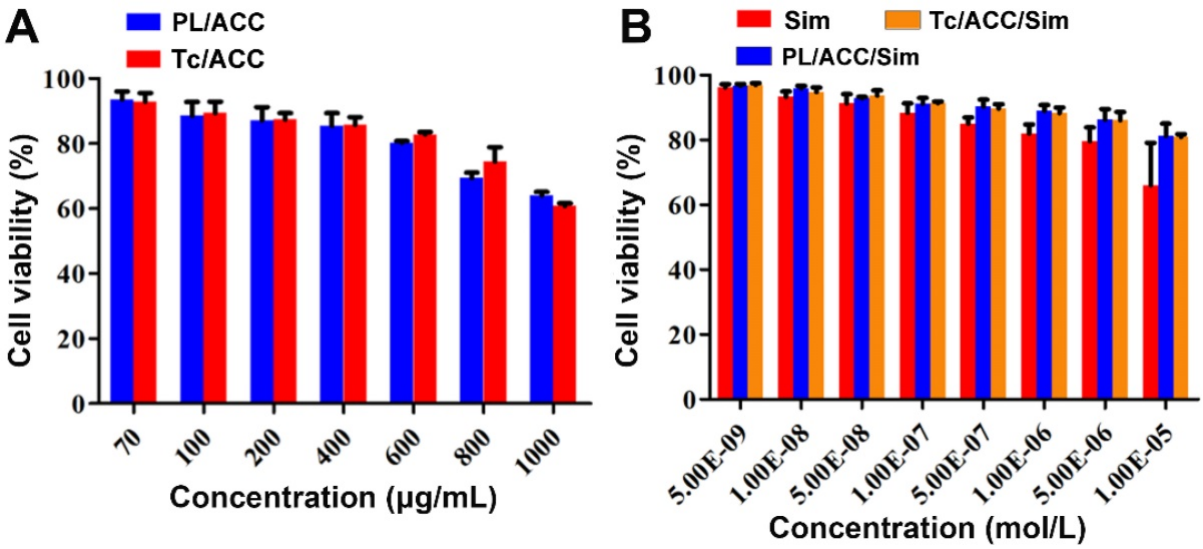
Optimization of administration dosage. MC3T3-E1 cells were exposed to a series of concentrations of PL/ACC, Tc/ACC, Sim, PL/ACC/Sim and Tc/ACC/Sim for 48 h. Afterwards, the cell viability influence of (**A**) Sim free carrier (PL/ACC and Tc/ACC) and (**B**) Sim containing formulations were evaluated using MTT assay. Results were expressed as mean ± S.D. (*n* = 3).

In order to find the optimized administration dosage, the cytotoxicity of drug free carriers and DDSs on MC3T3-E1 cells were investigated individually. As shown in Figure 5A, both Sim free PL/ACC and Tc/ACC showed low cytotoxicity on cells with 50% cellular growth inhibition (IC_50_) value of 4428.3 ± 208.8 μg/mL and 3754.3 ± 88.3 μg/mL (n=3), respectively. The reduce in IC_50_ in Tc/ACC might related to the antibiosis nature of Tc, which demonstrated that the hybrid nanocarriers using naturally originated PL and ACC exerted high biocompatibility on cells and were suitable for the safe delivery of drugs. Afterwards, the Sim loaded DDSs were tested under different given drug concentrations to explore the optimal concentration of Tc/ACC/Sim for osteoblast differentiation and mineralization. As shown in Figure 5B, all Sim containing groups showed a concentration dependent toxic effect on MC3T3-E1 cells. However, it was noted that Sim loading using the hybrid carrier composed of PL and ACC significantly reduced the cytotoxicity induced by Sim, especially when the concentration of Sim was higher than 1×10^-6^ mol/L, which was in line with previous reports that DDS can effectively reduce the side effects of loaded drugs 27, 32. Considering the cytotoxicity effect of Tc on MC3T3-E1 cells, the comparative cytotoxicity between PL/ACC/Sim and Tc/ACC/Sim was investigated. It was demonstrated that no significant cytotoxicity difference was observed between PL/ACC/Sim and Tc/ACC/Sim on MC3T3-E1 cells, suggesting that the modification of Tc under the tested concentrations exerted almost negligible cytotoxicity effect. Moreover, it was observed that when the concentration of Sim was higher than 1×10^-7^ mol/L, the cytotoxicity of cells in Sim group fell below 90%. As a result, in order to exclude cytotoxicity effects of Sim on the differentiation and mineralization of cells, the drug concentration at 1×10^-7^ mol/L was selected in the following experiments unless otherwise stated.

### *In vitro* osteoporosis reversing effect

**Figure 6:**
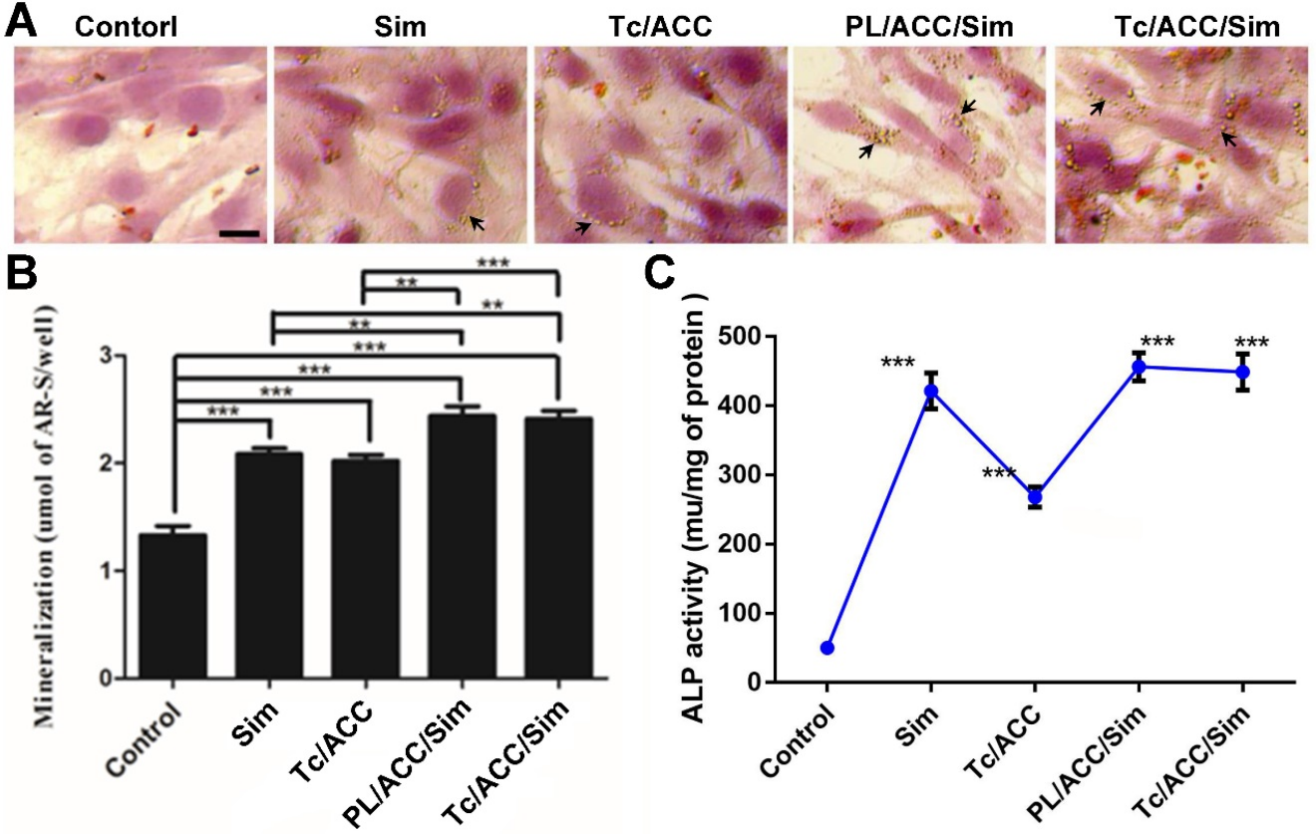
Effects of Sim-loaded nanoparticles on mineralized nodule formation and ALP activity in MC3T3-E1 cells. MC3T3-E1 cells were cultured in standard osteogenic differentiation and mineralization medium containing 10 mM β-glycerophosphate, 50 µg/mL ascorbic acid and different formulations at the equal Sim concentration of 10^-7^ M). Afterwards, the mineralization of the extracellular matrix was evaluated by optical microscopy and macroscopic observation (**A**), quantitative mineralization results (**B**) at 14 days post incubation, and stimulation of ALP activity (**C**) at 7 days post incubation. Results were expressed as mean ± S.D. (*n* = 3). *p < 0.05, **p < 0.01, ***p < 0.001, significant differences compared with the control.

In order to evaluate the osteoporosis reversing effect of Tc/ACC/Sim, its influence on osteoblast mineralization was firstly assessed by quantitative mineralization experiments. MC3T3-E1 cells were cultured in standard osteogenic differentiation and mineralization medium containing 10 mM β-glycerophosphate, 50 μg/mL ascorbic acid and different formulations at the equal Sim concentration of 1×10^-7^ mol/L for 14 days 33. As shown in Figure 6A, the increased mineralized nodules (indicated by arrows) can be observed in the group cultured with free Sim when compared to the control group, which might due to the potential of Sim to increase BMP-2 expression in osteoblasts and promote the differentiation and mineralization of osteoblasts through the BMP-Smad signaling pathway. On the other hand, it was noted that calcium supplement using Tc/ACC carrier also showed beneficial effects on osteoblasts than untreated group, which suggested the positive effect of calcium supplement in reversing osteoporosis. Further investigation using western blotting further confirmed that BMP-Smad signaling pathway was involved in this benefit (Figure S2) as a result of calcium supplement. In particular, the combination of calcium supplement and osteoblasts promotion using ACC and Sim (PL/ACC/Sim and Tc/ACC/Sim) showed further enhanced benefits on osteoblast mineralization. The quantitative analysis of alizarin red S staining was also performed and displayed in shown in Figure 6B. In line with results obtained in Figure 6A, the quantitative analysis also revealed that mono therapy using either calcium supplement or osteoblasts promotion could exert beneficial effects on osteoblast mineralization while the combination of both approaches could further elevated the final outcome, suggesting the promising synergetic effects of this strategy.

Sim has the potential to increase BMP-2 expression and promote the formation of new bone *in vitro*. ALP is an up-regulated enzyme with positive relation to the osteoblast differentiation, which was considered as a marker of new bone formation 34. As a result, we examined the expression of ALP in MC3T3-E1 cells treated with different formulations. As shown in Figure 6C, compared with control group, the Tc/ACC group showed certain elevation on the ALP activity, which further confirmed the positive effect of calcium supplement in osteoblast differentiation for osteoporosis therapy. Additionally, in line with previous reports, it was noted that all Sim containing groups could significantly increase the ALP activity in MC3T3-E1 cells 35, 36. In particular, the Sim-loaded DDSs showed comparable benefits to that of free Sim, which suggested that the loaded Sim was able to be effectively released from the DDSs to realize osteoblast promotion.

### *In vivo* osteoporosis reversing effect

**Figure 7:**
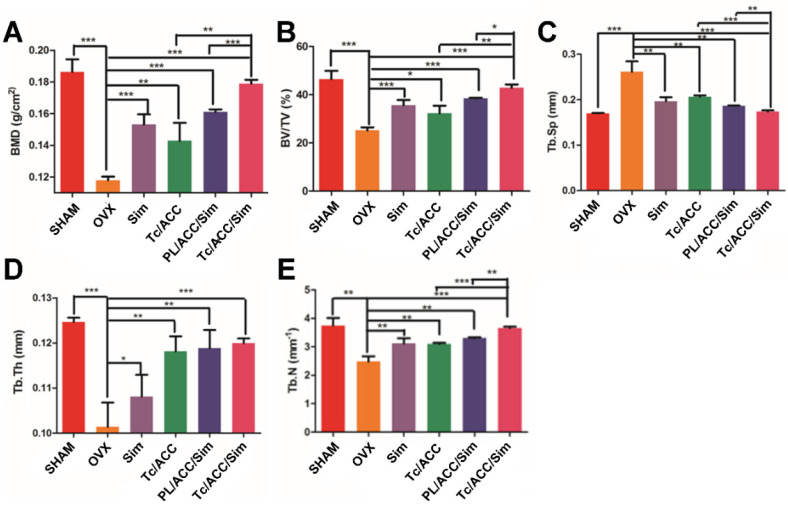
Female Sprague Dawley rats were randomly selected and subjected to sham operation or bilaterally oophorectomy. The treatment groups were intravenously administered with Sim, PL/ACC/Sim or Tc/ACC/Sim group at the Sim dose of 1 mg/kg/2 days for 2 months. Afterwards, the rats were sacrificed and the micro-CT analysis of bone tissue from each group were conducted. (**A**) Trabecular bone density (BMD); (**B**) Trabecular bone volume percentage (BV/TV); (**C**) Trabecular separation (Tb.Sp); (**D**) Trabecular thickness (Tb.Th) and (**E**) trabecular number (Tb.N) of the femur. Results were expressed as mean ± S.D. (*n* = 3). *p < 0.05, **p < 0.01, ***p < 0.001, significant differences compared with the control.

Micro-CT scanning was used to analyze the trabecular bone mineral density (BMD, Figure 7A), trabecular bone volume percentage (BV/TV, Figure 7B), trabecular separation (Tb. Sp, Figure 7C), trabecular thickness (Tb. Th, Figure 7D) and trabecular number (Tb. N, Figure 7E) at 12 weeks post-treatment. In Figure 7A, the femur BMD was distinctly different between the SHAM group and the OVX group, with a value of 0.186±0.007 g/cm^2^ in the SHAM group and 0.118±0.002 g/cm^2^ in the OVX group. The BMD of the OVX group was reduced by 36.56% compared to that in the SHAM group, indicating that the osteoporotic model rats were established successfully 37.

BMD and BV/TV are extensively used as predictors for evaluating osteoporosis 38. In our study, OVX treatment significantly decreased the BMD and BV/TV in rats, while mono therapy using either calcium supplement or osteoblasts promotion could significantly reverse this process. Previous reports have revealed that Sim can increase BMP-2 expression in osteoblasts and promote the formation of new bone through the BMP-Smad signaling pathway 39, 40, which was confirmed by the increased BMD and BV/TV in all Sim treated groups in our study. In particular, combination therapy (PL/ACC/Sim and Tc/ACC/Sim) further enhanced the effect with a better performance. Similar tendencies were also observed in Tb. Th and Tb. N groups when compared to the OVX group. Accordingly, OVX increased the Tb. Sp of treated subjects at the end of the test while the combination of calcium supplement and osteoblasts promotion could significantly reverse this process than applying mono therapy alone. These results indicated that Sim could stimulate bone formation by promoting osteoblast differentiation and mineralization, thereby repairing damaged bone tissue and the synergetic effects using calcium supplement could exert further beneficial effects on the therapy. Moreover, it was noted that Tc/ACC/Sim showed better performance than PL/ACC/Sim in all experiments, which provided decisive evidence to prove the promising bone targeting of Tc/ACC/Sim and its positive effects in osteoporosis therapy.

**Figure 8:**
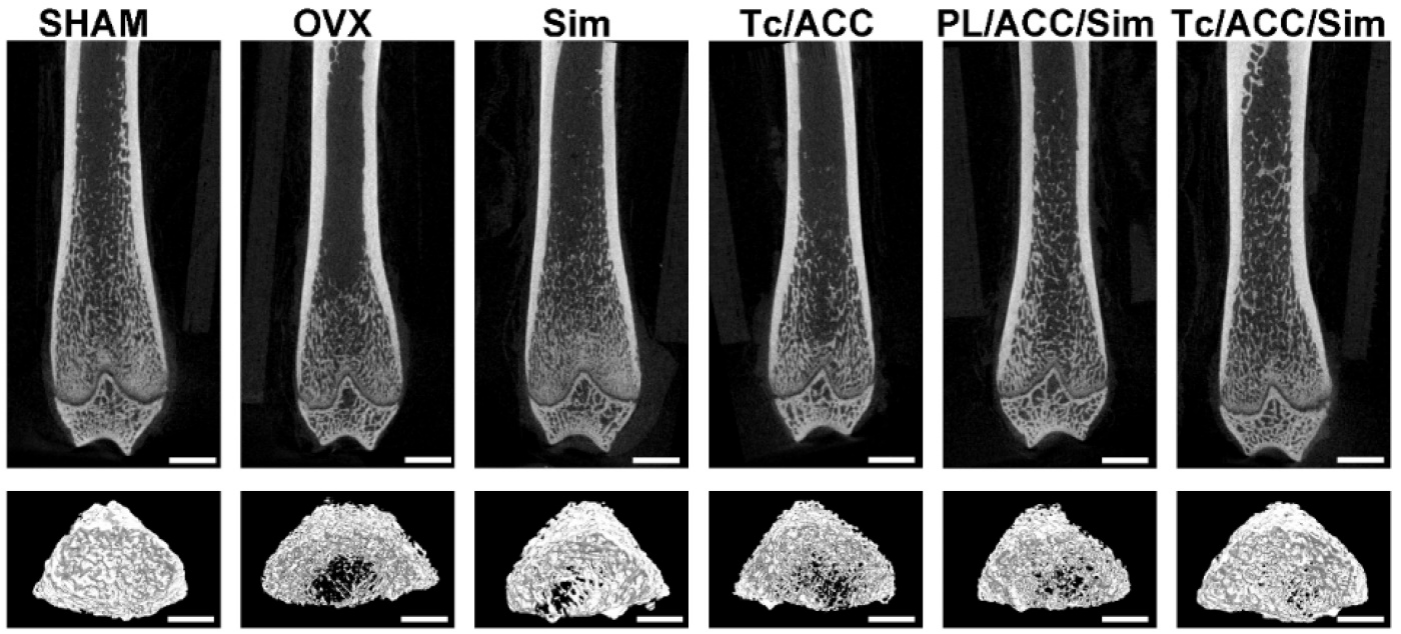
The 2D and 3D images of trabecular bone measured by micro-CT. Scale bar: 1mm.

The 2D images and 3D images of trabecular bone were also measured by micro-CT to confirm the above mentioned conclusion. As shown in Figure 8, OVX resulted in significant trabecular bone loss in the subjects, which resulted in osteoporosis. In line with above results, both calcium supplement and osteoblast promotion significantly alleviated the osteoporosis and increased the compact bone substance as compared to OVX group, indicating a significant improvement in microstructure and bone volume. As expected, the synergetic effects of calcium supplement/osteoblast promotion and Tc modification (Tc/ACC/Sim group) further increased the health of trabecular bone which achieved the best microstructure and bone volume in the micro-CT assay.

**Figure 9:**
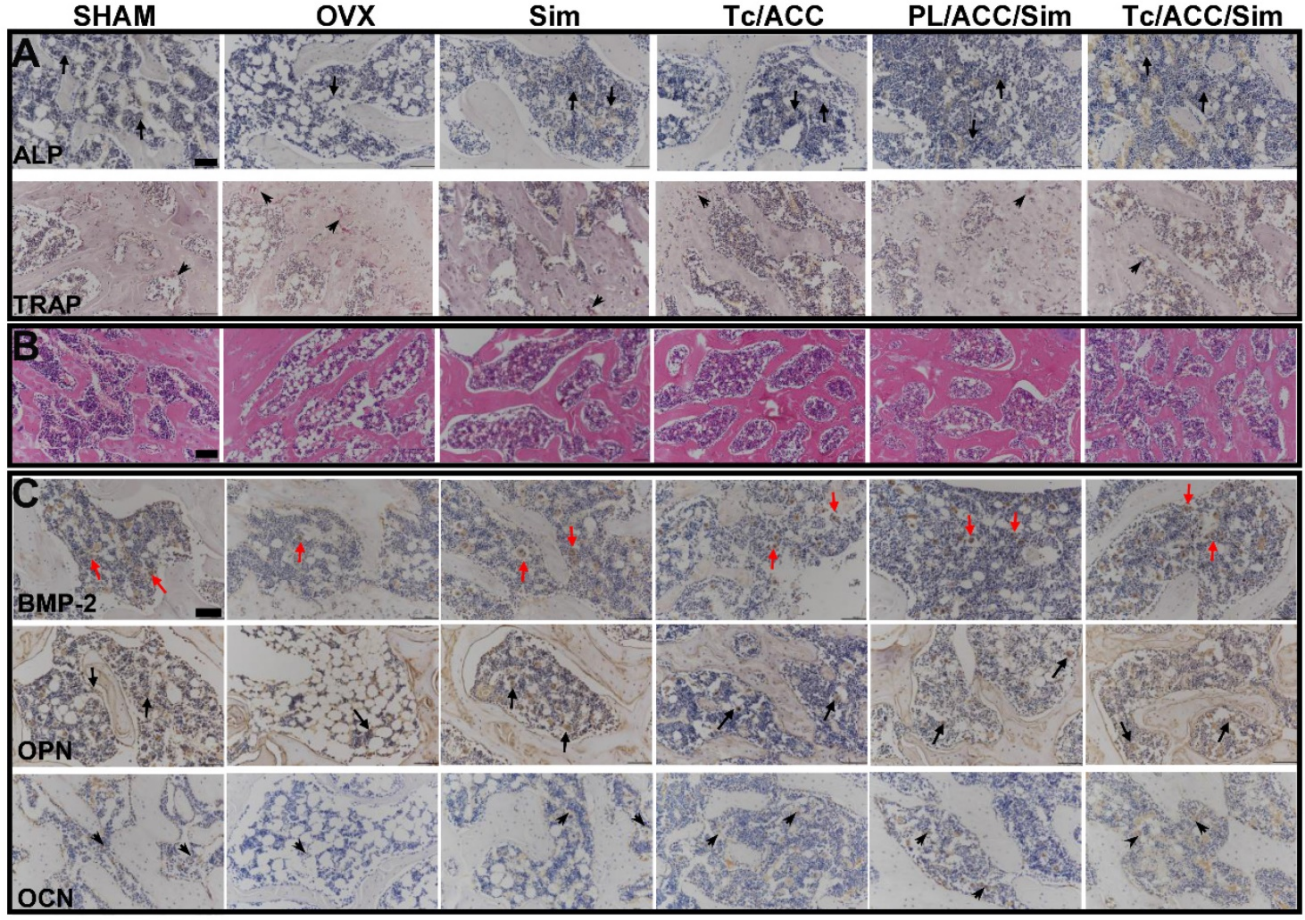
(**A**)ALP activity (arrows) and TRAP assay results (arrowheads) of bone tissue sections. (**B**) Histological assessment of bone formation in each group using HE staining. (**C**) Immunohistochemical staining for BMP-2 in typical newly-formed bone tissue (red arrows) and immunohistochemical staining for the osteogenic markers OPN (arrows) and OCN (arrowheads). Scale bars = 100 µm.

During the process of BMP-2-induced osteoblast differentiation, Runx2 expression and ALP activity increase as the result of BMP-Smad signaling pathway activation 41. As shown in Figure 9A, in either ACC or Sim containing groups, the activity of ALP was higher than that of the OVX group. In detail, several cells were brought together, and the surrounding gray area suggested the newly formed bone matrix where the activity of ALP was high, indicating that new bone was forming. These results were also confirmed by the mean optical density (MOD) studies in Figure S3. As a result, it was inferred that the beneficial effects of calcium supplement in reversing osteoporosis were also partially attributed to the BMP-2-induced osteoblast differentiation 42, which finally resulted in synergistic osteoblast promotion with Sim for better osteoporosis therapy. As expected, the calcium supplement (using ACC) or osteoblast promotion (using Sim) significantly reduced the TRAP activity than that in OVX group. Most importantly, in line with above assays, in both experiments, the combination of calcium supplement and osteoblast promotion achieved the better performance than mono therapy. In addition, the modification of Tc could increase the distribution of Tc/ACC/Sim in the bone and increase the concentration of Sim at the bone site. Therefore, the ALP activity of the Tc/ACC/Sim groups was the highest and almost the same as that of the SHAM group. Accordingly, the TRAP activity of Tc/ACC/Sim was as low as that of the SHAM group 43.

In order to further confirm the above conclusions, all sections of femurs were evaluated histologically (Figure 9B and Figure S4). As displayed in Figure 9B, normal compactness of the femur and competent trabeculae were observed in the SHAM group, while the OVX group showed sparse loss of interconnectivity and thinning of the trabeculae, thereby showing widened intertrabecular spaces. The OVX group showed more adipose tissue than the other groups did. There was a statistically significant increase in trabecular interconnectivity in the all Sim containing groups in comparison to the OVX group. Moreover, the trabecular bone was much more prominent in the Tc/ACC/Sim group than that in PL/ACC/Sim due to the bone-targeting ability of Tc, thus increasing the accumulation of Sim in bones and increasing the drug efficacy. It is also noted that the Tc/ACC/Sim treated group showed more resemblance to the SHAM group. Overall, the bone histological analysis indicated a remarkable recovery effect of Sim and showed a restored architecture with this treatment regime in the ovariectomy-induced osteoporosis model in rats 44.

In order to confirm that BMP-Smad signaling pathway was involved in the osteoporosis reversing process of calcium supplement and to further determine the synergistic benefits of calcium supplement on the osteoblast promotion with Sim, the IHC staining of different groups was performed and shown in Figure 9C and Figure S5. Sim could promote the differentiation and mineralization of osteoblasts through the BMP-Smad signaling pathway, therefore, we can study the efficacy of Sim by studying the expression of BMP-2 protein and the mineralization markers OPN and OCN. As shown in Figure 9C, compared to that in OVX group, all three markers were significantly evaluated in Tc/ACC group. As expected, these three markers were also highly expressed in all Sim containing groups. In particular, the Tc/ACC/Sim group integrated Tc targeting and synergetic therapy showed the most satisfying performance (expression of BMP-2, OPN and OCN) among all formulations which was comparable to that of SHAM group. These results indicated that calcium supplement in combination with Sim could preferably induce synergistic osteoblastic differentiation through the BMP-Smad signaling pathway and the DDS using Tc as the targeting moiety could specifically transport more drugs to the bone for better osteoporosis therapy 45.

## Conclusion

In this study, we developed a novel Tc modified ACC carrier (Tc/ACC) for the delivery of Sim (Tc/ACC/Sim). The obtained DDS was composed of nanosized particles which showed high biocompatibility on MC3T3-E1 cells. Moreover, Tc modification could enhance the accumulation of the DDS to bones both *in vitro* and *in vivo*. In addition, *in vitro* and *in vivo* osteoporosis reversing assays demonstrated beneficial effects of mono therapy using either calcium supplement or Sim induced osteoblast promotion. Most importantly, the combination of both approaches showed synergistic effects with much more elevated osteoblastic differentiation through the BMP-Smad signaling pathway. Moreover, the Tc modification also potentiated this effect *in vivo* though its bone targeting nature. In summary, the Tc/ACC/Sim integrated Tc targeting and synergetic therapy showed the most satisfying performance both *in vitro* and *in vivo*, which might offer a novel approach for the effective reversing of osteoporosis.

## Supplementary Material

Supplementary figures.

## Abbreviations

ACC: Amorphous calcium carbonate
Tc: Tetracycline
HAP: Hydroxyapatite
BMPs: Bone morphogenetic proteins
TFG: Transforming growth factor
MMSC: Marrow mesenchymal stem cells
Sim: Simvastatin
DDS: Drug delivery system
PL: Phospholipid
SA: Stearic acid
DAPI: 4',6-Diamidino-2-phenylindole
FITC: Fluorescein isothiocyanate
DSC: N,N'-disuccinimidyl carbonate
ODA: Otcadecylamine
EDC: 1-(3-dimethylaminopropyl)-3-ethylcarbodiimide
HOBT: 1-Hydroxybenzotriazole
MTT: 3-(4,5-dimethylthiazol-2-yl)-2,5-diphenyl-tetrazolium bromide
BCA: Bicinchoninic acid
ALP: Alkaline phosphatase
ICG: Indocyanine green
αMEM: Alpha-minimum essential medium
^1^H NMR: Hydrogen Nuclear magnetic resonance
DLS: Dynamic light scattering
TEM: Transmission electron microscope
HPLC: High-performance liquid chromatography
DL: Drug loading
FBS: Fetal bovine serum
OVX: Ovariectomized
BMD: Bone mineral density
BV/TV: Bone volume fraction
Tb. Th: Bone tickness
Tb. N: Bone number
Tb. Sp: Bone separation
TRAP: Tartrate-resistant acid phosphatase
HE: Hematoxylin and eosin
ICH: Immunohistochemistry
OPN: Osteopontin
OCN: Osteocalcin
