# Supplementary material for: Calcium Supplement by Tetracycline guided amorphous Calcium Carbonate potentiates Osteoblast promotion for Synergetic Osteoporosis Therapy
Source: Theranostics. 2020 Jul 9;10(19):8591–605. doi: 10.7150/thno.45142 (PMC7392017; doi:10.7150/thno.45142)
Supplement: Supplementary file 1 — Supplementary figures. [file thnov10p8591s1.pdf]

Calcium supplement by tetracycline guided amorphous calcium carbonate potentiates osteoblast promotion for synergetic osteoporosis therapy

Jianwei Wang [b, 1], Shan Tao [a, 1], Xiangyu Jin [a], Yanqing Song [a], Wentao Zhou [a], Haiya Lou [c], Rui Zhao [c], Cheng Wang [a,d,] *, Fuqiang Hu [a], Hong Yuan [a,] *

[a] *College of Pharmaceutical Science, Zhejiang University, 866 Yuhangtang Road, Hangzhou, 310058, China*

[b] *The Second Affiliated Hospital, School of Medicine, Zhejiang University, No. 88 Jiefang Road, Hangzhou, 310009, China*

[c] *Sir Run Run Shaw Hospital, School of Medicine, Zhejiang University, No. 3 Qingchun East Road, Hangzhou, 310016, China*

[d] *School of Pharmaceutical Engineering & Life Science, Changzhou University, Changzhou, 213164, China*

* Corresponding author. E-mail: 11519016@zju.edu.cn; yuanhong70@zju.edu.cn;

1 These authors contributed equally to this work.

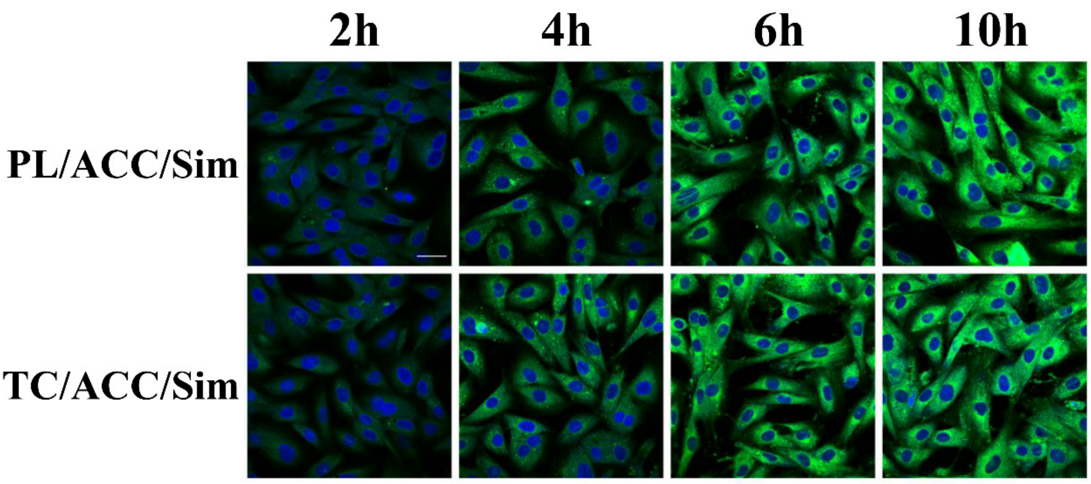

Figure S1. *In vitro* cellular uptake of PL/ACC/Sim and Tc/ACC/Sim. Scale bar = 30 μm.

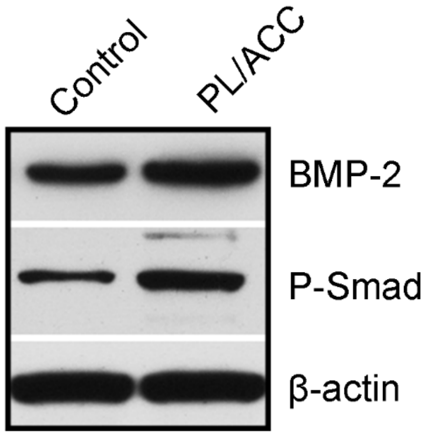

Figure S2. Western blot assay of BMP-Smad promotion efficacy of PL/ACC.

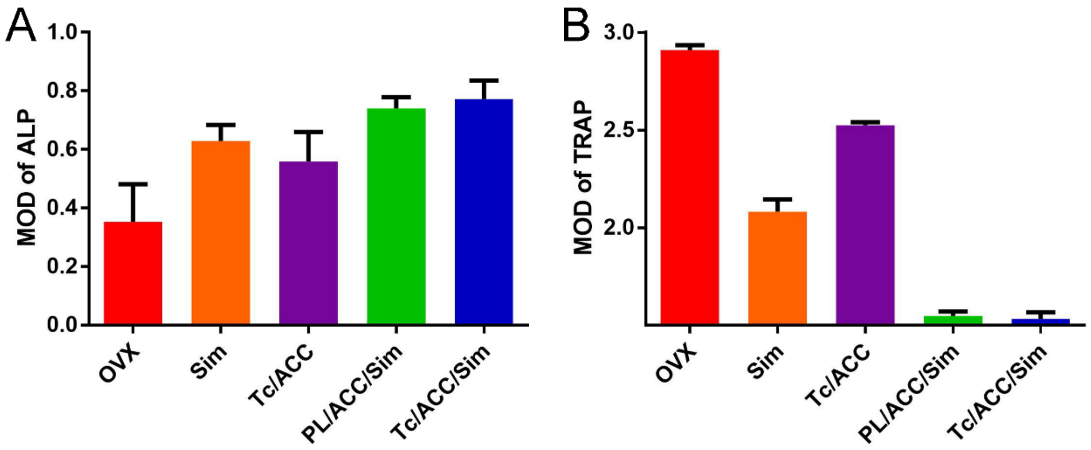

Figure S3. The calculated mean optical density (MOD) of ALP (A) and TRAP (B) corresponding to results in Figure 9. Results were expressed as mean ± S.D. (n = 3).

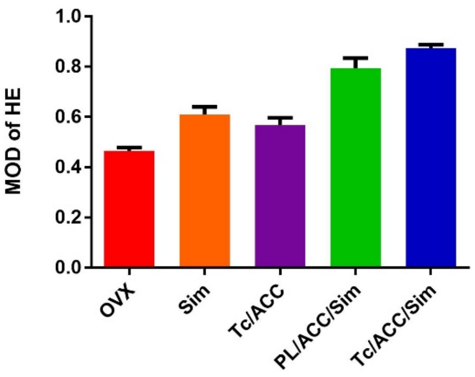

Figure S4. The calculated MOD of HE corresponding to results in Figure 10. Results were expressed as mean ± S.D. (n = 3).

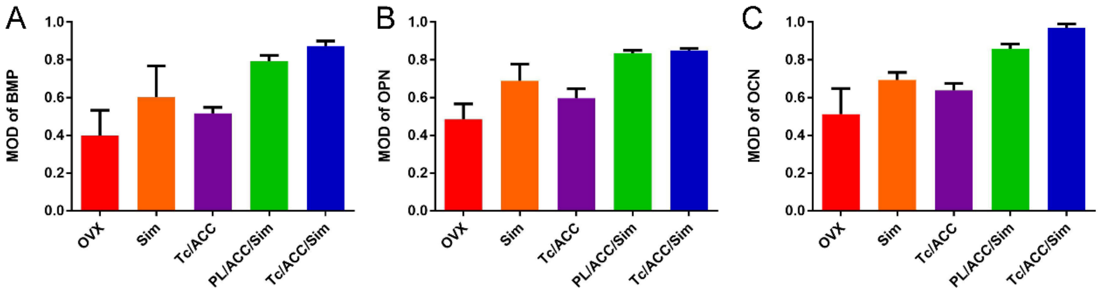

Figure S5. The calculated MOD of BMP-2 (A), OPN (B) and OCN (C) corresponding to results in Figure 11. Results were expressed as mean ± S.D. (n = 3).
